# Diagnostic utility of GeneXpert MTB/RIF assay versus conventional methods for diagnosis of pulmonary and extra-pulmonary tuberculosis

**DOI:** 10.1186/s12866-021-02210-5

**Published:** 2021-05-13

**Authors:** Asmaa Mohammed Elbrolosy, Rana H. El Helbawy, Osama M. Mansour, Reda Abdel Latif

**Affiliations:** 1 Medical Microbiology and Immunology Department, Faculty of Medicine, Menoufia University, Shebin el Kom, Egypt; 2 Chest Department, Faculty of Medicine, Menoufia University, Shebin el Kom, Egypt; 3 Chest Hospital, Ministry of Health and Population, Shebin el Kom, Egypt; 4 Public Health Department, Faculty of Medicine, Menoufia University, Shebin el Kom, Egypt

**Keywords:** Mycobacterium tuberculosis, GeneXpert MTB/RIF assay, Rifampicin

## Abstract

**Background:**

Tuberculosis (T.B) is one of the major infectious diseases in the developing countries. The diagnosis of extrapulmonary T.B (EPTB) remains problematic and emergence of resistant strains poses a significant threat. Improved diagnosis of tuberculosis is a global priority for proper control. The study aimed to assess the diagnostic accuracy of GeneXpert MTB/RIF assay for diagnosis of pulmonary TB (PTB) and EPTB and to evaluate the performance of GeneXpert system for demonstrating rifampicin resistance among the studied patients.

**Methods:**

A total of 582 clinical samples (449 pulmonary; 430 sputum and 19 bronchoalveolar lavage (BAL) and 133 extra-pulmonary origins; 26 pleural fluid, 62 CSF, 19 ascetic fluid, 12 pus and 14 urine) were collected from patients under clinical and radiological assessment of either PTB or EPTB who were admitted to Menoufia Chest Hospital over a period of three years. Clinical samples were processed and investigated for detection of *Mycobacterium tuberculosis* (MTB) by both Xpert assay and the conventional methods including Ziehl-Neelsen (ZN)/acid-fast bacillus (AFB) smear microscopy and Lowenstein-Jensen (LJ) culture. Patients′ demographic, clinical characteristics and risk factors for acquiring rifampicin resistance were analyzed.

**Results:**

The sensitivity, specificity, false- negative rate and total accuracy of AFB smear microscopy respectively were 72.1 %, 81.3 %, 27.9 and 78.8 % for PTB. However for EPTB, they were 63.2 %, 70.5 %, 36.8 and 68.4 % respectively in relation to LJ culture as the gold standard. GeneXpert MTB/RIF revealed better performance for PTB than EPTB. For PTB, it showed 90.2 % sensitivity, 86.9 % specificity, and 9.8 % false- negative rate. For EPTB, the assay showed a sensitivity of 81.6 %, specificity of 78.9 % and false- negative rate of 18.5 %. Multivariate regression analysis showed that presence of EPTB and contacts with known TB cases were independent risk factors for developing rifampicin resistance.

**Conclusions:**

GeneXpert MTB/RIF assay is a rapid and highly sensitive technique for diagnosis of PTB or EPTB. Its simplicity and accuracy make this new method a very impressive tool for diagnosis of MTB and rifampicin resistance.

## Background

Tuberculosis remains a global health burden especially in developing countries. In 2019, an estimated 10 million people fell ill with TB worldwide, a total of 1.4 million people died from TB (including 208 000 people with HIV). Worldwide, TB is one of the top 10 causes of death and the leading cause from a single infectious agent (above HIV/AIDS) [1].

EPTB comprises 25 % of all TB cases all over the world, and even higher levels in HIV-infected individuals and children [2]. The diagnosis of EPTB remains challenging for both the clinicians and microbiologists. Nevertheless, the difficulty to gain access to specific sampling sites results in paucibacillary samples and reduces the sensitivity of conventional diagnostic tools [3].

Egypt is classified as a middle/low-level country according to TB prevalence. The estimated TB annual prevalence is 11/100 000 cases with smear-positive active PTB, and 24/100 000 cases with all types of TB [4].

The elimination of TB requires early, rapid and accurate diagnosis and treatment. Acid-fast bacilli microscopy with ZN staining is the most frequent laboratory technique used for MTB diagnosis. However, limitations associated with microscopy lead to misdiagnosis [5]. Although the technique is rapid and inexpensive, its sensitivity is variable (20–80 %) [6].

Bacteriological culture is the gold standard technique for diagnosis of TB, which can also provide testing for drug resistance. Likewise, such techniques require complex laboratory infrastructure and takes long time to get results. In fact, most personnel who need culture to diagnose their TB will not have access to the test results in time to save their lives or to avoid transmission to others [7].

Emergence of drug resistance is a worrisome problem during anti-tuberculous therapy. Point mutations in an 81-bp rifampicin resistance determining region of the *rpoB* gene (RRDR) have been detected in more than 90 % of rifampicin-resistant TB strains. The effect of certain mutations in different codons varies substantially in different countries [8].

Multidrug-resistant tuberculosis is a type of TB that is resistant to at least the two main first-line anti-TB drugs; rifampicin and isoniazid. Patients become infected with MDR-TB either when they are exposed to a resistant strain or when improper treatment leads to selection of a resistant strain [8]. Annually, approximately 3.3 % of new TB patients and approximately 20 % of previously treated patients become infected with MDR-TB, leading to higher mortality rates [9].

In low income countries, it is becoming increasingly difficult to treat MDR-TB. Treatment modalities are limited and expensive, and are not always available. Extensively drug-resistant TB (XDR-TB) is a form of MDR- TB with extra resistance to more TB drugs that therefore responds to even fewer available anti-TB agents. It has been declared in 117 countries worldwide (WHO) [10].

The employment of rapid molecular methods for the diagnosis of MTB is considered to be a significant asset by WHO for the diagnosis and monitoring of tuberculosis disease. GeneXpert MTB/RIF system is a fully automated real-time semi-nested PCR assay was endorsed as the most rapid test for diagnosis of PTB by WHO [11]. The new molecular techniques bring a considerable gain in the diagnosis of EPTB [12].

The main objectives of the current study were to comprehensively evaluate the diagnostic accuracy and clinical utility of GeneXpert MTB/RIF assay for diagnosis of both PTB and EPTB versus the standard conventional methods involving ZN smear microscopy and mycobacterial culture and to analyze the sociodemographic criteria of the studied cases. Data concerning rifampicin resistance were also correlated with the patients’ risk factors.

## Methods

This cross-sectional study was carried out during the period from 1st October 2017 to the last of September 2020 (three years interval) at the Bacteriology Laboratory of Shebin Elkom Chest Hospital, Menoufia governorate, Egypt in collaboration with the Medical Microbiology & Immunology and Chest Departments of Faculty of Medicine, Menoufia University during which a total of 449 pulmonary samples [430 sputum and 19 bronchoalveolar lavage (BAL)] and 133 extrapulmonary samples (26 pleural fluid, 62 CSF, 19 ascetic fluid, 12 pus and 14 urine) were respectively collected from patients with PTB and EPTB after clinical and radiological assessment (one sample from each participant). Samples were either from culture-proved/confirmed cases, new cases or from patients with treatment failure or relapse.

### Ethics statement

The study protocol was approved by the local ethics Committee of Faculty of Medicine, Menoufia University Hospitals and all the patients and/or their guardians have signed informed consent. The overall refusal rate was 9.5 %.There were no demographical or clinical differences between recruited cases and those who refused to participate.

### Collection of pulmonary and extrapulmonary samples

Four hundred-thirty of the recruited cases with suspected PTB provided three consecutive early morning sputum samples over a 2-day period. For patients who were unable to expectorate the sputum, ultrasonic nebulizer technique was used for sputum induction. Another 19 BAL samples were also collected. For patients with suspected EPTB; pleural fluid, CSF, ascetic fluid, pus and urine samples were collected in sterile containers, held at 4^°^C until processed by standard laboratory procedures and the Xpert assay. All the collected specimens were processed as follows:

- Acid- Fast Bacillus (AFB) smear microscopy

Ziehl-Neelsen staining was performed on the first non-decontaminated sputum samples as well as BAL, pleural fluid, CSF, ascetic fluid, pus and urine sediments (liquid samples were first concentrated for 15min at 3000rpm and sediments were used). Purulent sputum was liquefied with N acetyl-L-cysteine as a mucolytic agent to increase the homogeneity of the sample before smear preparation. Smears were examined to explore the presence of acid-fast bacilli and graded as per the International Union against Tuberculosis and Lung Disease scale; negative for TB, scanty, + 1, +2, and + 3. A patient was considered positive if a minimum of one smear was graded scanty or higher. Culture-positive colonies were subjected to ZN staining to establish their acid-fast status [13].

- Culture on Lowenstein-Jensen (LJ) Media

Second sputum samples were decontaminated using N-acetyl-L-cysteine and sodium hydroxide (NALC-NaOH) (Petoff’s method) [14]. Subsequently, the decontaminated sputum and sediments of BAL, pleural fluid, CSF, ascetic fluid, pus and urine were inoculated onto LJ slants that were incubated aerobically at 37^o^C for 2–8 weeks. Cultures were not discarded as negative except after 8 weeks. The grown isolates were identified as MTB using the standard biochemical tests, including production of niacin, nitrate reduction and catalase [15].

The third unprocessed sputum and other extrapulmonary samples were collected in specialized containers and tested directly using the GeneXpert assay.

### GeneXpert MTB/RIF diagnostic system (Cepheid, Sunnyvale, CA, USA)

#### Principle of the assay

It is a fully automated cartridge–based molecular system that integrates sample processing, nucleic acid amplification and recognition of the target sequences. The assay uses nucleic acid probes that identify and report the presence or absence of the normal, rifampicin-susceptible, sequence of the *rpoB* gene of MTB. Five different colored beacons are used, each covering a separate nucleic acid sequence within the amplified *rpoB* gene. The results of the assay are: **a-** TB positive rifampicin resistant, **b-** TB positive rifampicin non resistant, **c-** TB not detected and **d-**Invalid result [5].

#### Procedure and sample preparation

One ml un-concentrated specimens (without centrifuge) were used for Xpert MTB/RIF assay. Sample reagent (two volumes of 0.1M NaOH and 0.1M isopropanol) was added in a 2:1 ratio to unprocessed specimen in falcon tube and the tube was manually agitated twice during a 15min incubation period at room temperature. Subsequently, 2ml of the inactivated sample was transferred to the test cartridge by a sterile disposable pipette (provided with kits).Cartridges were labeled by the specimen ID and loaded into The Xpert MTB/RIF instrument. The cartridge contains the wash buffer, reagents for DNA extraction and PCR amplification, and fluorescent probes to do the assay automatically [16].

An interviewer-administered structured questionnaire was used to collect primary data from the recruited cases, and a checklist for data collection from the clinical records. Univariate and multivariate logistic regression analyses were performed to assess the potential risk factors for the existence of rifampicin resistance.

### Statistical analysis

The data were collected, tabulated, and analyzed by SPSS (statistical package for social science) version 20.0 on IBM compatible computer(SPSS Inc., Chicago, IL, USA).Categorical data was described as number and percentage and compared using Chi square and fisher’s Exact test accordingly. Quantitative data was described as mean, standard deviation and range, Shapiro Wilk test of normality was performed to check normality of the data, and it was analyzed by using Mann Whitney U test (not normally distributed data), diagnostic accuracy of AFB smear and GeneXpert MTB/RIF assay in relation to LJ culture as a gold standard, Receiver operating characteristics (ROC) curve was drawn to explore area under the curve of GeneXpert MTB/RIF assay. Multivariate regression analysis using binary logistic regression was used for independent risk factors for rifampicin resistance.

## Results

This study was conducted on 582 TB cases collected during the period from 1st October 2017 to the last of September 2020; they were 449 (77.1 %) PTB and 133 (22.9 %) EPTB cases. Age, sex and residence showed no significant difference between both TB types; rate of contact with other TB cases was 50.1 % & 47.4 % in PTB & EPRB respectively with no significant difference between them while the rate of retreated cases was significantly higher among EPTB than in PTB (19.5 % versus 5.3 % respectively: *P* ˂0.001) (Table 1).

**Table 1:** Socio-demographic, clinical and laboratory data of the studied cases

**Parameters**	**Patients with PTB(*n* =449)**	**Patients with EPTB(*n* = 133)**	**Test**	***P* value**
**Age (years)**				
Mean ± SD	50.56±15.65	50.17±16.69	0.18	0.86
Range	1 – 77	2 – 77		
**Age groups**				
0 – 15	5 (1.1%)	4 (3.0%)	7.06	0.07
16 – 35	122 (27.2%)	38 (28.6%)		
36 – 60	229 (51.0%)	54 (40.6%)		
>60	93 (20.7%)	37 (27.8%)		
**Gender**				
Male	333 (74.2%)	93 (69.9%)	0.94	0.33
Female	116 (25.8%)	40 (30.1%)		
**Residence**				
Rural	368 (82.0%)	105(78.9%)	0.61	0.43
Urban	81 (18.0%)	28 (21.1%)		
**Contact with a known TB patient**				
Yes	225 (50.1%)	63 (47.4%)	0.31	0.58
No	224 (49.9%)	70 (52.6%)		
**Previous anti-TB treatment**				
New case	425 (94.7%)	107 (80.5%)	26.36	<0.001**
Retreated case	24 (5.3%)	26 (19.5%)		

** Highly significant statistical difference; Quantitative data was analyzed using Mann Whitney U test, Qualitative data was analyzed using Chi square test (***X***^**2**^)

AFB smears demonstrated positivity in 33.2 % (149/449) and 39.1 % (52/133) of PTB & EPTB cases respectively. On using LJ culture, 122/449 cases (27.2 %) with PTB and 38/133 cases (28.6 %) with EPTB were positive but with no significant difference (Table 2). Considering LJ culture as the gold standard, AFB smear revealed sensitivity of 72.1 %, specificity of 81.3 %, false- negative rate of 27.9 % and total accuracy of 78.8 % for PTB. However for EPTB, the sensitivity, specificity, false- negative rate and total accuracy of AFB respectively were 63.2 %, 70.5 %, 36.8 and 68.4 % (Table 3).

**Table 2:** Comparison between PTB and EPTB regarding laboratory diagnostic methods (AFB smear, LJ culture and Gene Xpert MTB/RIF assay)

**Laboratory methods**	**PTB(*n* = 449)**	**EPTB(*n* = 133)**	***X***^**2**^	***P*value**
**AFB smear**				
Negative	300 (66.8%)	81 (60.9%)	2.22	0.70
** +**	40 (8.9%)	12 (9.0%)		
** ++**	31 (6.9%)	10 (7.5%)		
** +++**	44 (9.8%)	18 (13.5%)		
** ++++**	34 (7.6%)	12 (9.0%)		
**AFB smear**				
Negative	300 (66.8%)	81 (60.9%)	1.59	0.21
Positive	149 (33.2%)	52 (39.1%)		
**LJ culture**				
Positive	122 (27.2%)	38 (28.6%)	0.10	0.75
Negative	327 (72.8%)	95 (71.4%)		
**Gene Xpert MTB/RIF assay**				
Invalid	42 (9.4%)	11 (8.3%)	1.55	0.46
Positive	111 (24.7%)	40 (30.1%)		
Negative	296 (65.9%)	82 (61.7%)		
**Rifampicin resistance**	**(*n* = 111)**	**(*n* = 40)**		
Yes	14 (12.6%)	13 (32.5%)	7.92	0.005*
No	97 (87.4%)	27 (67.5%)		

X^2^ Chi square test

*Significant statistical difference

**Table 3:** AFB smear microscopy performance for PTB and EPTB in relation to LJ culture as the reference standard

**AFB smear microscopy**	**LJ Culture**			
	**PTB** (***n* = 449)**		**EPTB (*n* = 133)**	
	**Positive (*n* = 122)**	**Negative (*n* = 327)**	**Positive (*n* = 38)**	**Negative (*n* = 95)**
**Positive**	88	61	24	28
**Negative**	34	266	14	67
**Sensitivity**	72.1%		63.2%	
**Specificity**	81.3%		70.5%	
**PPV**	59.1%		46.2%	
**NPV**	88.7%		82.7%	
**False -positive rate**	18.7%		29.5%	
**False- negative rate**	27.9%		36.8%	
**Accuracy**	78.8%		68.4 %

**Table 4:** GeneXpert MTB/RIF diagnostic system performance for PTB and EPTB in relation to LJ culture as the reference standard

**Specimen type**	**PTB (*n* = 449)**		**EPTB (*n* = 133)**					**PTB**	**EPTB**
	**Sputum**	**BAL**	**Pleural fluid**	**CSF**	**Ascetic fluid**	**Pus**	**Urine**		
**No.**	430 (95.8 %)	19 (4.2 %)	26 (19.5 %)	62 (46.6 %)	19 (14.3 %)	12 (9.0 %)	14 (10.5 %)	449	133
**True- positive**	106	4	7	12	5	3	3	110	31
**False- positive**	0	1	2	4	1	0	1	1	9
**True- negative**	272	12	13	40	11	5	7	284	75
**False negative**	12	0	2	1	1	2	2	12	7
**Invalid or error**	40 (9.3 %)	2 (10.5 %)	2 (7.7 %)	5 (8.1 %)	1 (5.3 %)	2 (16.7 %)	1 (7.1 %)	42 (9.4 %)	11 (8.3 %)
**Sensitivity**	89.8%	100%	77.8%	92.3%	83.3%	60.0%	60.0%	90.2%	81.6%
**Specificity**	87.2%	80.0%	76.5%	81.6%	84.6%	71.4%	77.8%	86.9%	78.9%
**PPV**	100 %	80.0%	77.8%	75.0%	83.3%	100%	75%	99.1%	77.5%
**NPV**	95.8	100%	86.7%	97.6%	91.7%	71.4%	77.8%	95.9%	91.5%
**False- positive rate**	0.0%	20%	11.8%	10.2%	7.7%	0.0%	11.1%	0.3%	9.5%
**False -negative rate**	4.2%	0%	22.2%	0%	16.7%	40.0%	40.0%	9.8%	18.5 %

**Fig. 1:**
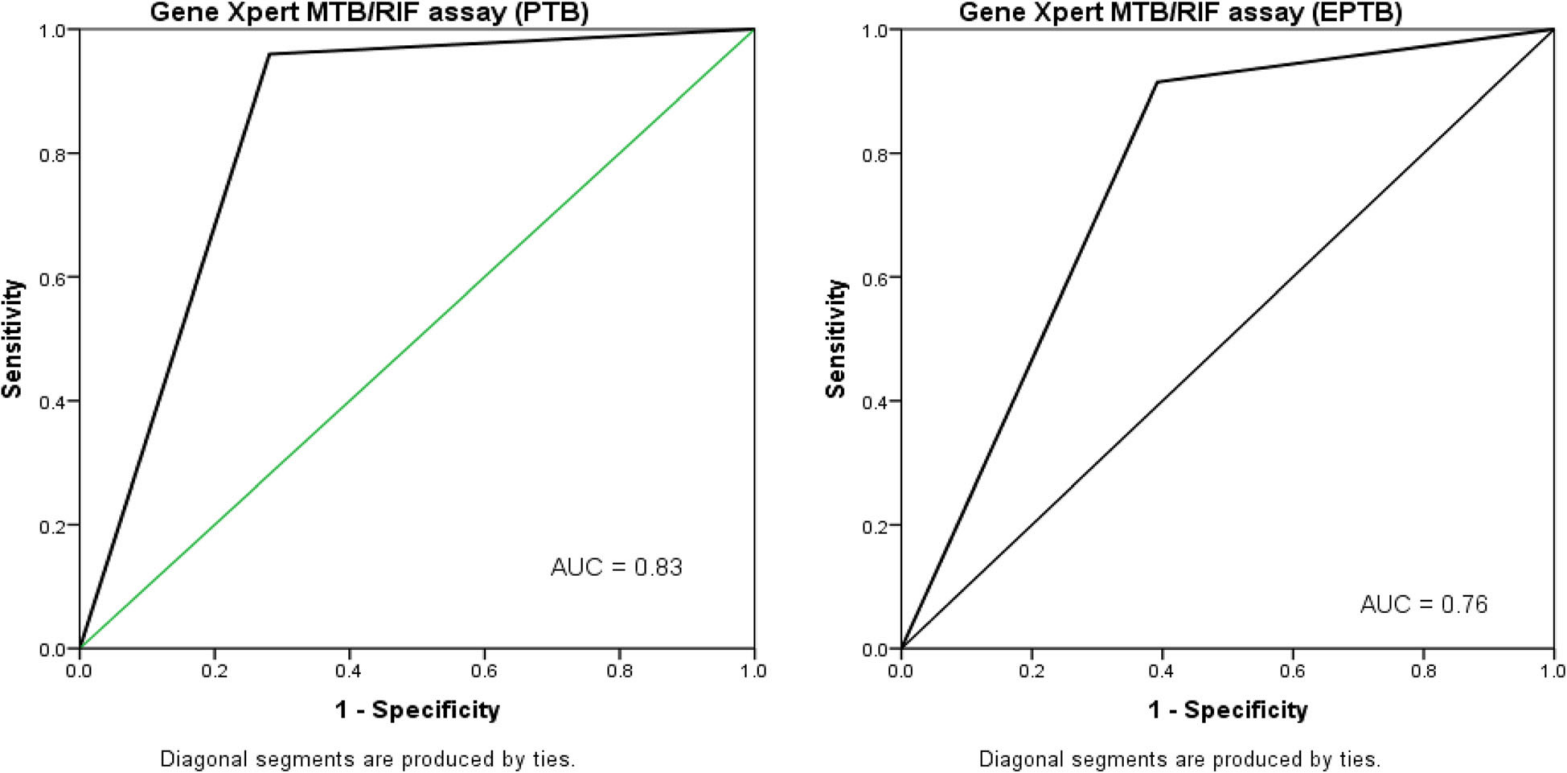
**a** Roc curve analysis for GeneXpert MTB/RIF assay versus LJ culture in PTB cases, **b** Roc curve analysis for GeneXpert MTB/RIF assay versus LJ culture in EPTB cases

GeneXpert MTB/RIF assay demonstrated positivity in 24.7 % (111/449) and 30.1 % (40/133) for PTB & EPTB cases respectively. However, the assay proved to be invalid in 9.4 % (42/449) and 8.3 % (11/133) of PTB & EPTB samples respectively with no significant difference. According to GeneXpert assay, rifampicin resistance was detected in 12.6 % (14/111) and 32.5 % (13/40) of PTB & EPTB cases respectively with a significant statistical difference (P ˂0.005) (Table 2). ROC curve analysis for diagnostic performance of the Xpert assay in PTB and EPTB demonstrated area under the curve of 0.827 & 0.755 respectively (Fig. 1).

GeneXpert MTB/RIF diagnostic performance showed better measurement for detection of PTB than in EPTB. For PTB, it showed sensitivity of 90.2 %, specificity, of 86.9 % and false- negative rate of 9.8 % while for EPTB, the assay revealed 81.6 % sensitivity, 78.9 % specificity and 18.5 % false- negative rate (Table 4).

Table 5 showed that, negative smear, history of previous TB treatment and previous contact with known TB case were risk factors for developing rifampicin resistance and binary logistic regression analysis for these significant factors revealed that TB type (EPTB) and contact with a known TB case were independent risk factors for developing rifampicin resistance (P = 0.004 & 0.03 and odds ratio of 4.21 & 2.85 respectively).

**Table 5:** Univariate and multivariate regression analysis showing the socio-demographic, behavioral and clinical factors associated with multidrug-resistant TB in patients with PTB and EPTB

**Variables**	**Patients infected with rifampicin- resistant strains (*n* = 27)**		**Patients infected with rifampicin- susceptible strains (*n* = 124)**		***P*-value**
**Age (years)**					U
** Mean **±SD	49.44±14.88		48.72±14.81		0.82
** Range**	15 – 77		15 – 77		
**Age groups**					
0 – 15	1 (33.3)		2 (66.7)		0.73
16 – 35	6 (13.3)		39 (86.7)		
36 – 60	15 (19.5)		62 (70.5)		
>60	5 (19.2)		21 (80.8)		
**Gender**					
Male	23 (19.0)		98 (81.0)		0.47
Female	4 (13.3)		26 (86.7)		
**Residence**					FE
Rural	26 (21.0)		98 (79.0)		0.049
Urban	1 (3.7)		26 (96.3)		
**Smoking**					
Positive	19 (22.4)		66 (77.6)		0.10
Negative	8 (12.1)		58 (87.9)		
**TB type**					
▪ PTB	14 (12.6)		97 (87.4)		0.005*
▪ EPTB	13 (32.5)		27 (67.5)		
Positive Smear					
▪ Yes	14 (13.2)		92 (86.8)		0.02*
▪ No	13 (28.9)		32 (71.1)		
**Previous TB treatment**					FE
▪ Yes	20 (19.4)		114 (80.6)		0.02*
▪ No	7 (41.2)		10 (58.8)		
**Contact with a known TB Patient**					
▪ Yes	18 (24.7)		55 (75.3)		0.035*
▪ No	9 (11.5)		69 (88.5)		
**HIV results**					
Unknown	11 (15.9)		58 (84.1)		0.39
Positive	2 (40.0)		3 (60.0)		
Negative	13 (17.1)		63 (82.8)		
**Residence **	1.95	1.07	0.07	2.110.07	1.11 – 3.15
	**Multivariate regression analysis**				
	B	SE	*P* Value	Odds ratio	95% CI
**TB type **	1.41	0.49	0.004	4.21	2.15 – 5.48
**Smear **	0.10	0.48	0.83	0.91	0.56 – 1.22
**Contact with TB case **	1.05	0.49	0.03	2.85	1.19 – 7.88

*U* Mann Whitney U, *FE* Fisher's Exact test, *Significant statistical difference

## Discussion

Tuberculosis is still a public health threat with an increasing death rate especially in low resource settings. Early detection and starting proper treatment is ultimately important to reduce the mortality rate. The diagnosis of EPTB represents a serious problem and current tests are of limited accuracy [17].

Acid-fast bacillus smear microscopy and culture are the cornerstones for TB diagnosis. Although considered as the gold standard method, culture is time-consuming, requires proper infrastructure and technical expertise [18]. Likewise, the AFB smear, is rapid and inexpensive, however, its sensitivity is variable (20–80 %) and cannot differentiate between MTB and non- tuberculous mycobacteria (NTM) because of limited specificity [6]. In the context of such limitations, the fully automated Xpert MTB/RIF assay was endorsed by the WHO as the most rapid test for diagnosis of PTB [11].

The priority of Xpert MTB/RIF as a diagnostic method of MTB is attributed to its suitability and feasibility as quick, reliable, controllable, effortless, and economic test [19]. The GeneXpert uses a DNA PCR technology for concurrent detection of MTB and rifampicin resistance- related mutations [5].

The present study enrolled a total of 582 patients, 449 with PTB and 133 with EPTB from those admitted to the inpatient wards or attending the outpatients’ clinics of Menoufia General Chest Hospital. Our main targets were to compare the diagnostic accuracy of Xpert MTB/RIF diagnostic yield method with the AFB smear microscopy and culture tests as reference standards for diagnosis of both PTB and EPTB. The incidence and risk factors for acquiring rifampicin resistance were also investigated.

Importantly, EPTB cases represent 22.9 % of the cases under survey. There are multiple Egyptian studies that documented the rate of EPTB in different locations ranging from 21 % for Hibah [20] in El-Behira governorate to about 37.3 % for Sobh et al. [21] in Aswan governorate. Higher rate (64.14 %) was reported in the study conducted by El Bouhy et al. [22] in Assiut chest hospital. Such observations highlight the need for comparative study between different Egyptian locations to interpret this difference.

In this work, the majority of EPTB cases were in the active age (15–60) of male gender (69.9 %). Of all the collected extrapulmonary samples, CSF accounted for 46.6 % followed by pleural fluid (19.5 %). Hibah [20] found that EPTB was prevalent in males than females, in the middle age from 15 to 44 years old and that pleural site was the commonest, while Mohammadien et al. [23] found lower male affection (36.6 % ) and that lymph node & pleura were the commonest sites for EPTB. The differences may be owed to the different social and demographic characters in every studied area. Meanwhile, there is a need for more detailed survey to interpret the high rate of CSF samples.

According to the current results, the sensitivity and specificity of AFB smear microscopy were lower than those of GeneXpert MTB/RIF assay with either PTB (72.1 % vs. 90.2 %) or EPTB (63.2 % vs. 81.6 %) when using culture as the gold standard. Out of the 122 (27.2 %) true- positives samples that grew in culture, only 88/122 (72.1 %) were detected by AFB smear for PTB. As for EPTB samples, out of 38(28.6 %) true-positives samples, only 24/38 (63.2 %) were positives by AFB smears. These results came in parallel with previous studies conducted for evaluation of the Xpert assay performance [5, 24, 25]. The sensitivity of ZN smear may vary between different geographical regions and within the same regions between different laboratories, which is unlikely to occur with nucleic acid- based methods. ZN smear microscopy is still done to explore the degree of patients’ infectivity; the tuberculosis infectious dose is lower than ten bacilli. Meanwhile, the lower detection limit of AFB microscopy ranges from 5,000 to 10,000 AFB/ml; this means that AFB smear would miss many potentially infectious cases [26].

In a related matter, Muia et al. [27] documented a sensitivity of 81.8 % and specificity of 84.3 % for smear microscopy as compared to the reference culture method. Much lower results were generated from other laboratories that showed sensitivity ranging from 20 to 80 % and specificity of 74.5-80.7 % [28]. The remarkable variations in the sensitivity of AFB microscopy are related to various factors including: sample collection; smear preparation; slide examination; use of fluorescent versus conventional stains, administration previous anti-TB drugs and differences in the performance depending on the operator [29].

In our study, the false- negative rate of smear microscopy reached 27.9 % with PTB and 36.8 % with EPTB samples. Similarly, Meawed and Shaker 2016 confirmed that, ZN smear microscopy carries the risk of false-negative results and incompetency to discriminate between drug- susceptible and drug- resistant strains of MTB owing to poor sample quality coupled with a need for an experienced specialist. Meanwhile, culture being the gold standard, it proceeds for weeks up to months to yield results, and depends on sophisticated laboratory facilities and skilled technicians [30].

Although smear microscopy has the advantages of being rapid, simple and lower cost procedure, this study revealed that smear microscopy is prone to misdiagnosis MTB infection. Misdiagnosis of either PTB or EPTB has grave implications of continued transmission, higher mortality rates and delayed appropriate therapy [27].

For diagnosis of PTB, the GeneXpert MTB/RIF assay proved overall sensitivity, specificity, PPV and NPV of 90.2 %, 86.9 %, 99.1 and 95.9 % respectively in relation to LJ culture. Out of the 122 (27.2 %) isolates that grew in culture, 110 (90.2 %) were identified by GeneXpert as MTB and were true- positives for PTB. Gawish et al. 2019 [31] reported sensitivity, specificity, PPV & NPV of 100 %, 94.7 %, 91.7 % and100 % respectively for the Xpert assay. This performance was almost comparable to that reported by Fouda et al. [5] in Egypt who declared that, the sensitivity and specificity of Xpert diagnostic method were 100 and 75 % respectively, PPV was 95.5 % and NPV was 100 %. In Iran, the sensitivity, specificity, PPV and NPV of Xpert assay were found to be 95.5 %, 96.7 %, 83.8 %, and 99.1 % respectively for PTB [16]. In the same field, the sensitivity, specificity, PPV and NPV for GeneXpert were 97.7 %, 100 %, 100 and 98.9 % respectively for tubercle bacilli identification in Kenya [27].

In a recently published research article in Egypt by Hefzy et al. [4] the detected sensitivity and specificity of the GeneXpert assay were 78.3 and 99.1 %, respectively for PTB. However, for extra-pulmonary specimens, the sensitivity and specificity of the assay were 37.1 and 99 %, respectively. Moreover, the author confirmed that GeneXpert assay showed almost perfect agreement with the bacterial culture for TB diagnosis and that the diagnostic accuracy of the GeneXpert assay was high in ruling in, but not in ruling out of EPTB.

The first analytical study to validate the GeneXpert technology noted that the assay had 100 % sensitivity and specificity for diagnosis of PTB [32]. In a multicenter study involving Peru, Azerbaijan, South Africa and India reported an overall sensitivity of 97.6 % with 98.1 % specificity [24]. A meta-analysis of 16 GeneXpert assay studies revealed a pooled sensitivity of 90 % and specificity of 98 % [33].

The false- positive results detected by Xpert MTB/RIF from one patient with PTB whose BAL sample was culture negative, may be due to the presence of residual DNA of old dead organisms owing to previous history of TB or a sub-clinical relapse of the disease. False- positive outcomes yielded by Xpert assay could be one of justifications due to cross-contamination, added to the fact that GeneXpert can detect intact bacteria and cannot explore free DNA fragments [5].

As for EPTB, overall sensitivity, specificity, PPV and NPV of Xpert assay were 81.6 %, 78.9 %, 77.5 and 91.5 % respectively in relation to LJ culture. Allahyartorkaman et al. found 76.5 % sensitivity, 95.9 % specificity, 62 % PPV, and 97.9 % NPV for diagnosis of EPTB by Xpert MTB/RIF assay. The author declared that Xpert MTB/RIF assay proved to be highly sensitive, specific and comparable to standard conventional methods for the diagnosis of PTB. However, the sensitivity and specificity for EPTB specimens were highly variable [16].

As we compared the ability of smear microscopy with Xpert MTB/RIF assay in PTB detection, smear microscopy detected 88/122 MTB cases while Xpert assay detected 110/122 including all true -positive cases of smear microscopy plus 22 positive cases among subjects with smear- negative results. Thus Gene Xpert MTB/RIF out performed AFB microscopy and established a diagnosis of presumptive PTB for few cases with smear- negative TB, which came in accordance with other previous studies [15, 16].

According to current results, the false- negative specimens were higher with EPTB than PTB for the Xpert assay (18.5 % vs. 9.8 %).It is evidenced that, the GeneXpert technique had variable performance for different biological samples compared with the optimal sputum samples in PTB. Extrapulmonary samples like CSF, ascetic fluid and urine samples had lower bacillary burdens; the diagnostic efficacy are quite variables in different studies [34].

The higher cost of the Xpert assay must be compared to the benefit from avoiding poor sensitivity and specificity of AFB microscopy. To reduce the relative high cost of the assay, it is important to decrease the chance of getting failed or invalid test result. The present study found that an Xpert MTB/RIF- based strategy is more effective than smear- based one for both PTB EPTB as well as for evaluation of transmission potential of infected cases.

As for rifampicin resistance, our study included analytical data that highlighted the most significant risk factors for development of rifampicin resistance among the studied cases. The univariate analysis revealed that all of the site or type of TB, previous exposure to anti-TB therapy, a positive smear and a history of contact with a known TB case were statistically significant factors (P < 0.05).This observation was consistent with previous studies conducted elsewhere and indicated that previous exposure to anti-TB treatment might be the most significant risk for MDR-TB [35, 36].

The acquired rifampicin resistance can occur when there is a history of incomplete treatment regimens lasting at least 1 month [36]. Prior inappropriate anti-TB regimen only suppresses the growth of susceptible bacilli but has no effect on other resistant strains, leading to suitable conditions for the dominant multiplication of pre-existing drug-resistant mutants [37]. MDR-TB cases in this study may have experienced similar conditions of previous inadequate treatment that led to the occurrence of MDR-TB. Additionally, the association between contact with known TB patient and MDR-TB was significant factor as observed in several other studies that also supported the hypothesis that contact with a known TB patient is linked with rifampicin resistance due to exposure to resistant TB strains [35].

### Limitations

Lack of Mycobacteria growth indicator tube (MGIT) as a rapid liquid culture methods and the potential impact on the study, lack of evaluating the diagnostic precision of the GeneXpert assay on samples other than those tested in this study (e.g., blood samples) and finally shortage of studying the impact of the assay on patient’s outcomes; are the most recognized limitations of this study.

## Conclusions

The out performance of Xpert MTB/RIF detected in current work is in agreement with other researchers who established the diagnosis in a significant proportion of cases. Moreover, the relative gain and more case detection by means of Xpert recommend performing Xpert as the first diagnostic test, to avoid extraordinary work load. High sensitivity of GeneXpert MTB/RIF detected in the current work allows ruling out the disease with a high degree of confidence.

## Data Availability

All data generated or analyzed during this study are included in this published article.

## Abbreviations

T.B: Tuberculosis
EPTB: Extrapulmonary T.B
PTB: Pulmonary TB
MTB: *Mycobacterium tuberculosis*
ZN: *Ziehl-Neelsen*
LJ: *Lowenstein-Jensen*
AFB: Acid-fast bacillus
WHO: World Health Organization
XDR-TB: Extensively drug-resistant TB
MDR-TB: Multi-drug resistant TB
BAL: Bronchoalveolar lavage
CSF: Cerebrospinal fluid
